# Efficacy and Safety of SGLT2 Inhibitors in Pediatric Patients and Young Adults: A Systematic Review and Meta-Analysis of Randomized Controlled Trials

**DOI:** 10.1155/2024/6295345

**Published:** 2024-10-22

**Authors:** Rafael dos Santos Borges, Ana Flávia Conegundes, Luiza Haikal de Paula, Rodrigo Lara Santos, Samuel Norberto Alves, Raquel Amaral Machado, Isadora Bussolaro Viana, Ana Cristina Simões e Silva

**Affiliations:** ^1^Faculty of Medicine, Universidade Federal de Minas Gerais, Avenue Prof. Alfredo Balena, 190-Santa Efigênia, Belo Horizonte-MG, Belo Horizonte 30130-100, Minas Gerais, Brazil; ^2^Interdisciplinary Laboratory of Medical Investigation, Unit of Pediatric Nephrology, Faculty of Medicine, Universiade Federal de Minas Gerais, Avenue Prof. Alfredo Balena, 190-Santa Efigênia, Belo Horizonte-MG, Belo Horizonte 30130-100, Minas Gerais, Brazil

## Abstract

**Introduction:** In recent decades, an increase in the incidence of type 2 diabetes mellitus (T2DM) in children and adolescents has been observed. Pediatric-onset T2DM differs from the adult-onset form, particularly regarding the durability of glycemic control and earlier appearance of complications. However, the scarcity of approved treatments and comprehensive studies on T2DM management in youth persists. Ongoing clinical trials seek to ascertain the efficacy and safety of sodium-glucose cotransporter 2 inhibitors (SGLT2i) in patients aged between 10 and 24 years with T2DM. Therefore, we aimed to perform a meta-analysis exploring the efficacy and safety of SGLT2i in pediatric patients and young adults with T2DM.

**Methods:** We searched PubMed, Embase, Cochrane, and Web of Science for randomized controlled clinical trials on the efficacy and safety of SGLT2i in children, adolescents, and young adults with T2DM compared with placebo. Statistical analysis was performed using RevMan 5.4 and R statistical software 4.2.1. Heterogeneity was assessed with *I^2^* statistics.

**Results:** We included three studies totaling 334 patients followed for 37.79 weeks. Reduction in HbA_1_C (MD = −0.93; 95% CI = −1.36 to −0.49; *p*  < 0.0001; *I^2^* = 0%) was significantly higher in SGLT2i group compared with placebo. The proportion of patients requiring rescue or discontinuation of study medication due to lack of efficacy was statistically lower in SGLT2i group compared with placebo (RR = 0.64; 95% CI = 0.43–0.94; *p*= 0.02; *I^2^* = 0%). SGLT2i and placebo were similar in terms of any adverse event (RR = 1.10; 95% CI = 0.96–1.27; *p*= 0.17; *I^2^* = 0%), serious side effects (RR = 1.06; 95% CI = 0.44–2.57; *p*=0.90; *I^2^* = 0%), and individual adverse effects.

**Conclusion:** In children, adolescents, and young adults with T2DM, SGLT2i appears to be effective and safe for glycemic control.

## 1. Introduction

In recent decades, global reports have observed an increase in the incidence of type 2 diabetes mellitus (T2DM) in children and adolescents, associated with the rise in overweight and obesity in this age group [1–3]. In this context, a global estimate of the incidence of type 2 diabetes in children and adolescents for the year 2021 projected 41,600 new cases diagnosed worldwide [4]. Considering different regions, in the United States, for example, an analysis from 2002–2003 to 2011–2012 indicated a 7.1% annual increase in overall incidence rates of type 2 diabetes in young people [5]. Another example of this increase is a study conducted in Germany, which indicated a 5.8% annual rise in the prevalence of type 2 diabetes in children and adolescents from 2002 to 2020 [6]. This trend is accompanied by the premature onset of insulin resistance and a rapid decline in *β*-cell function in comparison to their adult counterparts [7, 8]. Pancreatic endocrine function is estimated to diminish by 20%–35% annually from the age of 10–19 years old and by 7%–10% annually in adults [9, 10]. Besides, puberty contributes to physiological insulin resistance in adolescents, but not in adults; and body mass index (BMI) in people from 10 to 17 years with T2DM is often higher than that seen in adults [11, 12]. Consequently, pediatric patients can be considered more challenging to achieving adequate glycemic levels and have a heightened risk of disease exacerbation, early cardiorenal complications, and premature dependence on exogenous insulin throughout the course of the disease [13–17].

In this context, meticulous glucose control in childhood is imperative to mitigate complications and enhance long-term quality of life [8, 18, 19]. However, the scarcity of approved treatments and comprehensive studies on T2DM management in youth persists due to challenges in patient recruitment and the accelerated disease progression relative to adult-onset T2DM [19–21]. People less than 24 years old are under-represented in clinical trials of T2DM. In a large study sponsored by the United States National Institutes of Health, more than 5000 patients with T2DM younger than 30 years were excluded [22]. Moreover, the subset of young adults (18–24 years old) is systematically excluded from pediatric trials [23, 24], even though the characteristics of T2DM in this subgroup are quite similar to those of patients with 10–17 years old [25].

For 25 years, metformin stood as the singularly approved T2DM medication by the United States Food and Drug Administration (FDA) and the European Medicines Agency (EMA) for individuals over 10 years of age with T2DM. This posed a therapeutic challenge for patients unresponsive or intolerant to metformin [19, 26]. Despite its continued status as the primary therapeutic choice in this demographic, the landscape shifted in 2019 with FDA and EMA approval of daily liraglutide [27, 28] and, subsequently, in 2021 (FDA) and 2022 (EMA), of once-weekly exenatide [29, 30]. Both are glucagon-like peptide 1 (GLP-1) receptor agonists administered via subcutaneous self-injections [31, 32]. Nevertheless, concerns over adherence to injectable medications persist in pediatric patients, emphasizing the persistent need for the development and approval of new oral drugs [19, 21].

Sodium-glucose cotransporter 2 inhibitors (SGLT2i) act by augmenting renal glucose excretion, thereby reducing filtered glucose reabsorption and plasma levels [33]. This drug class has improved cardiorenal outcomes in adults with T2DM, heart failure, and chronic kidney disease [34–37]. Consequently, ongoing clinical trials now seek to ascertain the efficacy and safety of SGLT2i in patients between 10 and 24 years old with T2DM [23, 24, 38]. Encouraging results of preliminary findings of the Tamborlane et al. [38] trial prompted the EMA to grant approval in 2021 to dapagliflozin for children 10 years old or older with T2DM, marking it the first orally administered therapy since metformin [39]. Later on, following similar results, the FDA and EMA granted approval in 2023 to empagliflozin [40, 41].

Currently, approved treatments for children and adolescents encompass daily oral metformin, subcutaneous insulin, subcutaneous GLP-1 receptor agonists (once-daily liraglutide and once-weekly exenatide), daily oral dapagliflozin, and daily oral empagliflozin. Canagliflozin, in turn, is limited to individuals aged 18 or older [40–42]. However, the restricted number of clinical trials requires a thorough data synthesis to comprehensively evaluate the safety and efficacy of SGLT2i treatment.

In light of these considerations, this study aimed to conduct a systematic review and meta-analysis to assess the potential benefits and risks associated with SGLT2i treatment in pediatric and young adult patients with T2DM.

## 2. Materials and Methods

The present systematic review and meta-analysis is registered in the International Prospective Register of Systematic Reviews (PROSPERO) [43] under the registration number CRD42023475140 and the Open Science Framework (OSF) [44] under the DOI https://doi.org/10.17605/OSF.IO/BK4TR. This systematic review with meta-analysis was executed in accordance with the methodological principles delineated in the Cochrane Handbook for Systematic Reviews of Interventions [45], and the findings are reported in accordance with the Preferred Reporting Items for Systematic Reviews and Meta-analyses (PRISMA) reporting guidelines [46].

### 2.1. Eligibility Criteria

The inclusion criteria for this systematic review with meta-analysis were (1) randomized controlled trials; (2) that directly compared SGLT2i versus placebo; (3) in pediatric patients and young adults with T2DM; and (4) published in the English language. Conversely, we excluded studies that were nonrandomized controlled trials, case reports or series, in vitro studies, animal studies, reviews, book chapters, opinions, conference abstracts, overlapping populations, or those that lacked a comparative analysis of SGLT2i outcomes. Furthermore, studies in patients with type I diabetes mellitus, older than 24 years, or using hypoglycemic medications other than metformin or insulin were also excluded.

### 2.2. Search Strategy and Data Extraction

The databases PubMed, EMBASE, Cochrane Library, and Web of Science were searched without time limits until June 2024. Our search strategy incorporated a combination of keywords and MESH terms, including: “SGLT2”, “SGLT-2”, “sodium-glucose cotransporter 2”, “sodium-glucose cotransporter-2”, “sodium-glucose co-transporter 2”, “sodium-glucose co-transporter-2”, “sodium-glucose transport protein 2”, “dapagliflozin”, “empagliflozin”, “canagliflozin”, “sotagliflozin”, “ipragliflozin”, “luseogliflozin”, “tofogliflozin”, “bexagliflozin”, “ertugliflozin”, “diabetes”, “diabetic”, “diabetal”, “diabetes mellitus”, “type 2 diabetes mellitus”, “type II diabetes mellitus”, “type 2 diabetes”, “type II diabetes”, “diabetes mellitus type 2”, “diabetes mellitus type II”, “diabetes type 2”, “diabetes type II”, “pediatric*⁣*^*∗*^”, “young”, “younger”, “children”, “child”, “infant*⁣*^*∗*^”, and “adolescent*⁣*^*∗*^”. The complete search strategy is presented in the supporting information. Additionally, we meticulously examined the reference lists from included manuscripts to identify any supplementary relevant studies. Two authors (R.S.B and A.F.C) independently extracted the data following predefined search criteria. Any discrepancy was resolved by discussion in a consensus meeting between the authors.

### 2.3. Endpoints

The efficacy endpoints under consideration included the mean change in BMI *z* score from baseline, mean change in HbA1c (%) from baseline, the percentage of patients achieving HbA1c < 7% at the end of the study, the mean change in fasting plasma glucose (FPG; mg/dL) from baseline, the proportion of patients requiring rescue or discontinuation of the study medication due to lack of efficacy, the change in systolic blood pressure (SBP; mmHg) from baseline, and the change in diastolic blood pressure (DBP; mmHg) from baseline. The safety outcomes comprised the rate of any adverse effects, serious adverse effects, adverse events leading to study discontinuation, any hypoglycemia event, severe hypoglycemia events, genital infections, ketoacidosis, headache, nasopharyngitis, vitamin D deficiency, and urinary tract infections (UTIs). The overall analysis of each endpoint was pooled based on two distinct time frames: short term (mean 25.57 weeks) and long term (mean 37.79 weeks), in order to assess if the effect of drug therapy was sustainable over time. The definition and classification of hypoglycemia adhered to the guidelines set forth by the American Diabetes Association (ADA) [47].

### 2.4. Risk of Bias Assessment

Two authors (I.B.V and R.L.S) separately performed the risk of bias and quality assessment of the studies using the Cochrane Collaboration tool for assessing the risk of bias in randomized studies (RoB v2.0) [48]. For each trial, the authors designated a score indicating “high risk,” “low risk,” or “unclear” in five domains: selection, performance, detection, attrition, and reporting bias. Specifically, we planned to evaluate the publication bias via the funnel plot asymmetry and Egger's test if the number of included studies was at least 10, as recommended in the Cochrane Handbook for Systematic Reviews of Interventions [45]. The quality of evidence was evaluated via the Grading of Recommendations, Assessment, Development, and Evaluations (GRADE) approach, considering aspects that may increase or decrease the quality of evidence [49]. The analyzed factors were study limitations, inconsistency, indirectness, imprecision, and publication bias. Ultimately, the final level of evidence was classified into high, moderate, low, and very low.

### 2.5. Statistical Analysis

Risk ratios (RR) alongside 95% confidence intervals (CI) were calculated to compare the occurrence of binary endpoints between the two groups, while continuous results were evaluated as mean differences (MD) with corresponding 95% CI. For the conversion of continuous data initially reported in the median (interquartile range), we utilized the estimated mean and standard deviation provided by Luo et al. [50] and Wan et al. [51], respectively. We used the inverse variance (IV) method for continuous outcomes and the Mantel–Haenszel (M–H) method for binary outcomes [45]. Cochran's *Q* test and *I^2^* statistics were employed to assess heterogeneity. Endpoints were categorized as having low heterogeneity if *p*  > 0.10 and *I^2^* <30%, moderate heterogeneity if *I^2^* fell between 30% and 75%, and high heterogeneity if *I^2^* exceeded 75%. Significance was attributed to *p* values <0.05. We used the random-effects model to account for potential variability among studies, which often yields a more realistic approach in real-world scenarios where study populations, interventions, and conditions differ, making it more suitable for generalizing results to different settings and populations. Additionally, this approach covers unobserved sources of variability and can better handle heterogeneity if it arises in future updates of the meta-analysis as more studies are included [45]. The statistical analyses were conducted using Review Manager 5.4 (Nordic Cochrane Centre, The Cochrane Collaboration, Copenhagen, Denmark) [52] and R statistical software version 4.2.1 (R Foundation for statistical computing) [53].

### 2.6. Sensitivity Analysis

We conducted a prespecified sensitivity analysis to evaluate the stability, robustness, and consistency of our findings regarding the change in HbA1c from baseline. The data were reassessed under the three following scenarios: (1) the mean change in HbA1c from baseline was evaluated in the intention-to-treat analysis, excluding data of rescue and/or treatment discontinuation; (2) the mean change in HbA1c from baseline was analyzed in the per-protocol analysis, excluding patients with relevant protocol deviations; and (3) we evaluated the proportion of patients with HbA1c ≥ 7% at baseline who achieved HbA1c < 7% at the end of the study.

### 2.7. Subgroup Analysis

Considering the main focus of this meta-analysis is pediatric patients, we presented data on the subgroup of children and adolescents (aged 10–17 years) for the outcomes of mean change in HbA1c from baseline and the rate of any adverse events. Moreover, since most of the current evidence on SGLT2 inhibitors in this population comes from the drug dapagliflozin, we also calculated data for these outcomes specifically for the dapagliflozin subgroup.

## 3. Results

### 3.1. Study Selection and Baseline Characteristics

**Figure 1:**
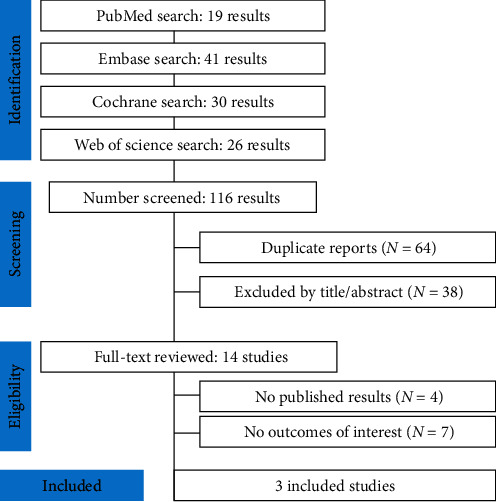
PRISMA flow diagram of study screening and selection.

As displayed in Figure 1, the search strategy initially yielded 116 articles. After removing 64 duplicates, an additional 38 manuscripts were excluded based on title and abstract screening because of the lack of the research question. Subsequently, the remaining 14 studies were fully reviewed. Among these, four were excluded due to the absence of results and seven for no outcomes of interest. Therefore, three published RCTs fully met all the inclusion criteria. The nonoverlapping population was 334 patients with T2DM. The overall cohort predominantly comprised White participants (54.07%) with a mean age of 14.88 ± 2.35 years. A significant majority, 94.31%, fell within the age range of 10–17 years. The average body weight was 86.97 ± 26.22 Kg, and the BMI was 31.92 ± 8.05 Kg/m^2^. Additionally, the mean SBP was 116.23 ± 12.29 mmHg, and the mean DBP was 72.10 ± 8.73 mmHg. Regarding diabetic parameters, the population exhibited a mean baseline HbA1c of 8.03 ± 1.43% and a mean FPG level of 157.61 ± 59.01 mg/dL. In terms of T2DM treatment, 51.50% of individuals were using metformin only, 10.48% were on insulin only, 36.53% were using both metformin and insulin, and 1.49% were not on any pharmacologic therapy (relying on diet and exercise due to metformin intolerance).  Table 1 provides an overview of the study characteristics and baseline demographics for the SGLT2i and placebo groups. Table S1 displays specific definitions used in individual studies.

### 3.2. Quality Assessment

The three included RCTs followed a similar design, with Tamborlane and DINAMO considered at low risk of bias in all domains of RoB2. T2NOW yielded an unclear overall risk of bias due to the randomization process and deviations from intended interventions (Table 2). Since we only included three RCTs in this meta-analysis, no formal assessment of the publication bias using the funnel plot asymmetry and Egger's test was recommended. The GRADE quality of evidence was deemed high for the long term of the following outcomes: mean change in HbA1c from baseline, mean change in FPG from baseline, and the proportion of patients requiring rescue or discontinuation of the study medication due to lack of efficacy (Table 3). The mean change in BMI *z* score from baseline in the long term was considered to be of very low certainty, downgraded due to (1) the unclear risk of bias in the T2NOW 2023 trial, which accounts for 55.2% of the weight of this outcome; (2) moderate heterogeneity (*I^2^* = 44%); and (3) the lack of statistical significance and wide CI of this outcome (Table 3). The long-term analysis of the proportion of patients achieving HbA1c < 7% at the study's conclusion was flagged as low certainty, downgraded due to the inclusion of patients with a baseline HbA1c < 7% (Table 3). Additionally, the rate of diabetic ketoacidosis (DKA) in the long term was considered to be of moderate certainty, downgraded due to the very limited sample size, which may have prevented the assessment of this relatively rare adverse event with reliable statistical power (Table 3).

### 3.3. Efficacy

**Figure 2:**
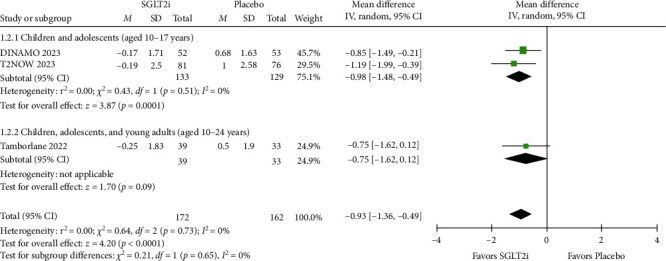
Mean change in HbA1c (%) from baseline (long follow-up).

**Figure 3:**
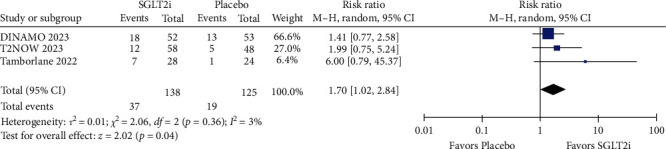
The proportion of patients achieving HbA1c < 7% at the end of the study (long follow-up).

**Figure 4:**
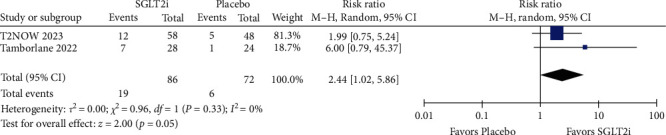
The proportion of patients with HbA1c ≥ 7% at baseline achieving HbA1c < 7% at the end of the study (long follow-up).

The mean change in HbA1c (%) from baseline had statistical significance with the use of SGLT2i compared to placebo in both the short follow-up period (MD = −0.94; 95% CI = −1.27 to −0.61; *p*  < 0.00001; *I^2^* = 0%; Figure S1) and the long follow-up (MD = −0.93; 95% CI = −1.36 to −0.49; *p*  < 0.0001; *I^2^* = 0%; Figure 2). In the sensitivity analysis, the superior effect of SGLT2i over placebo persisted in the short term, even after excluding data following rescue and/or treatment discontinuation (MD = −0.95; 95% CI = −1.36 to −0.55; *p*  < 0.00001; *I^2^* = 0%; Figure S2), and after excluding patients with relevant protocol deviations (MD = −1.13; 95% CI = −1.40 to −0.85; *p* =0.0033; *I^2^* = 0%; Figure S3). Specifically, the proportion of patients achieving HbA1c < 7% at the end of the study was significantly higher when using SGLT2i compared to placebo in the short term (RR = 2.03; 95% CI = 1.04–3.97; *p* =0.04; *I^2^* = 29%; Figure S4), and this outcome remained consistent in the long term (RR = 1.70; 95% CI = 1.02–2.84; *p* =0.04; *I^2^* = 3%; Figure 3). In the sensitivity analysis evaluating the proportion of patients with HbA1c ≥ 7% at baseline achieving HbA1c < 7% at the end of the study, the superior effect of SGLT2i was maintained in the short-term follow-up (RR = 3.15; 95% CI = 1.32–7.51; *p*=0.01; *I^2^* = 0%; Figure S5), but not in the long term (RR = 2.44; 95% CI = 1.02–5.86; *p* =0.05; *I^2^* = 0%; Figure 4).

**Figure 5:**
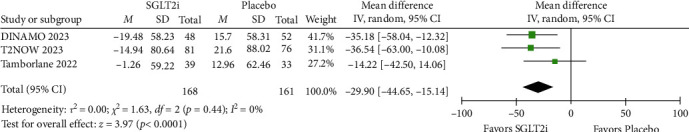
The mean change in FPG (mg/dL) from baseline (long follow-up).

Concerning the mean change in FPG (mg/dL) from baseline, a statistically significant reduction was observed in the SGLT2i group compared to placebo in both the short term (MD = −23.38; 95% CI = −36.30 to −10.46; *p* =0.0004; *I^2^* = 0%; Figure S6) and the long term (MD = −29.90; 95% CI = −44.65 to −15.14; *p*  < 0.0001; *I^2^* = 0%; Figure 5).

In assessing the proportion of patients requiring rescue or discontinuation of study medication due to lack of efficacy, no significant differences were observed between the SGLT2i group and the placebo group in the short term (RR = 0.46; 95% CI = 0.17–1.26; *p* =0.13; Figure S7) with moderate heterogeneity (*I^2^* = 34%). In the long term, the proportion was lower in the SGLT2i group (30/172; 17.44%) compared to the placebo group (44/162; 27.16%), indicating a more favorable profile in the SGLT2i group (RR = 0.64; 95% CI = 0.43–0.94; *p* =0.02; Figure S7) with a homogeneous pooled analysis (*τ*^2^ = 0; *X*^2^ = 0.3; df = 2; *p* =0.86; *I^2^* = 0; Figure S7).

**Figure 6:**
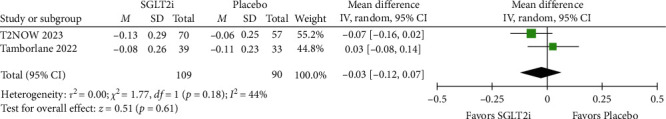
The mean change in BMI *z* score from baseline (long follow-up).

Concerning the weight control, we found no statistical differences on mean change in BMI *z* score from baseline between SGLT2i and placebo in both the short term (MD = −0.03; 95% CI = −0.11 to 0.06; *p* =0.52; *I^2^* = 46%; Figure S8) and long-term follow-up (MD = −0.03; 95% CI = −0.12 to 0.07; *p* =0.61; *I^2^* = 44%; Figure 6).

**Figure 7:**
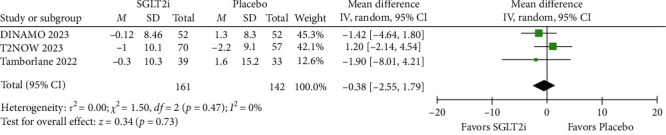
The mean change in SBP (mmHg) from baseline (long follow-up).

**Figure 8:**
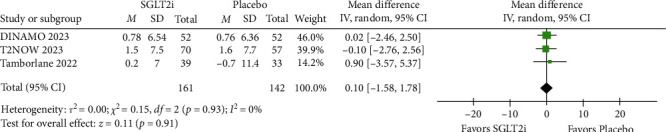
The mean change in DBP (mmHg) from baseline (long follow-up).

Regarding the change in blood pressure (mmHg) from baseline, no statistical differences were found between SGLT2i and placebo for SBP in both the short-term (MD = −0.94; 95% CI = −3.00 to 1.12; *p* =0.37; *I^2^* = 0%; Figure S9) and long-term follow-up (MD = −0.38; 95% CI = −2.55 to 1.79; *p* =0.73; *I^2^* = 0%; Figure 7). Similarly, DBP showed no significant differences between the groups in both the short term (MD = 0.17; 95% CI = −1.47 to 1.81; *p* =0.84; *I^2^* = 0%; Figure S10) and long term (MD = 0.10; 95% CI = −1.58 to 1.78; *p* =0.91; *I^2^* = 0%; Figure 8).

### 3.4. Safety

**Figure 9:**
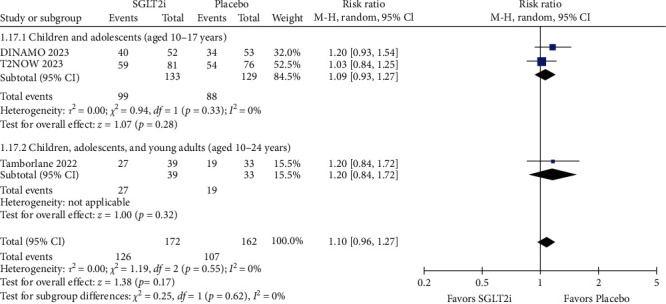
The rate of any adverse effect (long follow-up).

**Figure 10:**
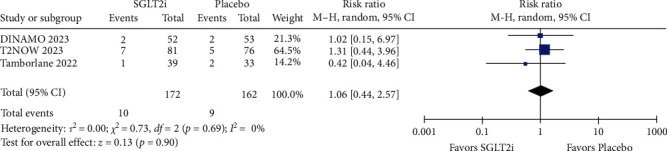
The rate of serious adverse effects (long follow-up).

The rate of any adverse effect was comparable between the SGLT2i and placebo groups in both the short term (SGLT2i 111/172 = 64.53%; placebo 100/162 = 61.73%; RR = 1.07; 95% CI = 0.86–1.33; *p*=0.53; *I^2^* = 40%; Figure S11) and long term (SGLT2i 126/172 = 73.25%; placebo 107/162 = 66.05%; RR = 1.10; 95% CI = 0.96–1.27; *p*=0.17; *I^2^* = 0%; Figure 9). Specifically evaluating the presence of serious adverse effects, there was no significant difference between the groups in the short-term (RR = 0.64; 95% CI = 0.18–2.25; *p*=0.48; *I^2^* = 0%; Figure S12) and long-term follow-up (RR = 1.06; 95% CI = 0.44–2.57; *p*=0.90; *I^2^* = 0%; Figure 10). Furthermore, the rate of adverse events leading to discontinuation of the study was similar between the SGLT2i and placebo groups in the short-term (RR = 1.07; 95% CI = 0.18–6.46; *p*=0.94; I^2^ = 0%; Figure S13) and the long-term (RR = 0.77; 95% CI = 0.14–4.25; *p*=0.76; *I^2^* = 0%; Figure S13).

Of particular interest, the rate of any hypoglycemia event was similar in the SGLT2i and placebo groups in both the short term (RR = 1.36; 95% CI = 0.77–2.38; *p*=0.29; *I^2^* = 38%; Figure S14) and long term (RR = 1.36; 95% CI = 0.83–2.24; *p*=0.22; *I^2^* = 27%; Figure S14). Specifically, severe hypoglycemia events were also not statistically different between the groups for both the short term (RR = 1.07; 95% CI = 0.24–4.79; *p*=0.93; *I^2^* = 12%; Figure S15) and long term (RR = 0.99; 95% CI = 0.20–5.04; *p*=0.99; *I^2^* = 27%; Figure S15).

Specifically, we observed no statistically significant difference between the SGLT2i and placebo groups, regardless of the follow-up time, in terms of genital infections, DKA, headache, nasopharyngitis, vitamin D deficiency, and UTIs (Figures S16–S21).

### 3.5. Subgroup Analysis

For the subgroup of children and adolescents (aged 10–17 years old), the mean change in HbA1c (%) from baseline had statistical significance with the use of SGLT2i compared to placebo in both the short follow-up period (MD = −0.97; 95% CI = −1.33 to −0.62; *p*  < 0.00001; *I^2^* = 0%; Figure S1) and the long follow-up (MD = −0.98; 95% CI = −1.48 to −0.49; *p*=0.0001; *I^2^* = 0%; Figure 2). Besides, the rate of any adverse effect was comparable between the SGLT2i and placebo groups in both the short-term (RR = 1.03; 95% CI = 0.76–1.40; *p*=0.85; *I^2^* = 65%; Figure S11) and long-term (RR = 1.09; 95% CI = 0.93–1.27; *p*=0.28; *I^2^* = 0%; Figure 9).

Concerning the subgroup of dapagliflozin, the mean change in HbA1c(%) from baseline had statistical significance with the use of SGLT2i compared to placebo in both the short follow-up period (MD = −0.97; 95% CI = −1.36 to −0.59; *p*  < 0.00001; *I^2^* = 0%; Figure S22) and the long follow-up (MD = −0.99; 95% CI = −1.57 to −0.40; *p*=0.0009; *I^2^* = 0%; Figure S22). Besides, the rate of any adverse effect was comparable between the SGLT2i and placebo groups in both the short-term (RR = 1.00; 95% CI = 0.74–1.36; *p*=0.98; *I^2^* = 47%; Figure S23) and long-term (RR = 1.06; 95% CI = 0.90–1.26; *p*=0.49; *I^2^* = 0%; Figure S23).

## 4. Discussion

In this systematic review and meta-analysis, the efficacy and safety of SGLT2i compared to placebo were evaluated in children and young adults with T2DM. The main findings were (1) SGLT2i and placebo had similar impacts on BMI *z* score in the short and long term; (2) SGLT2i had statistically significant benefits in HbA1c reduction in the short and long term; (3) SGLT2i accounted for a statistically higher proportion of patients achieving HbA1c < 7% at the end of the study in the short and long term, but its superiority was not maintained in the long term in patients with HbA1c ≥ 7% at baseline; (4) SGLT2i had statistically significant benefits in FPG reduction in the short and long term; (5) SGLT2i had statistically lower proportion of patients requiring rescue or discontinuation of study medication due to lack of efficacy in the long term; (6) SGLT2i and placebo had statistically similar effects on SBP and DBP in the short and long term; (7) SGLT2i and placebo showed no significantly differences in terms of any adverse event, severe side effects, and individual adverse effects.

SGLT2 is a protein situated in Segments 1 and 2 of the initial proximal convoluted tubules of the nephron. The protein exhibits low affinity, but high capacity, playing a pivotal role in reabsorbing over 90% of the filtered glucose [54–58]. In individuals with T2DM, there appears to be an upregulation of this transporter, leading to reduced glycosuria [58, 59]. Consequently, manipulating glucose transport through this protein has emerged as a promising therapeutic approach to address hyperglycemia [58, 59].

Considering that there are few studies including children, adolescents, and young adults for evaluation, the present systematic review and meta-analysis, after a criterious methodology, selected three Phase III trials that investigated the efficacy and safety of SGLT2i in patients 10–24 years old with T2DM. In a study conducted by Tamborlane et al. [38] (NCT02725593), empagliflozin 10 mg was compared to a placebo in a population aged 10–24 years over 24 weeks. The change in HbA1c concentration was not significant in the intention-to-treat analysis, but the per-protocol sensitivity revealed a superior effect of SGLT2i without safety concerns. In the DINAMO trial (NCT03429543), led by Laffel et al. [23], the objective was to assess the efficacy and safety of empagliflozin 10/25 mg in patients aged 10–17 years over a period of 26 weeks. Compared with the placebo, the group receiving SGLT2i exhibited a statistically significant reduction in HbA1c, with a similar safety profile between the groups. In the T2NOW trial (NCT03199053), conducted by Shehadeh et al. [24], dapagliflozin 5/10 mg and 5 mg saxagliptin were compared to a placebo in children and adolescents aged 10–17 years over a period of 52 weeks. The group receiving SGLT2i had a significant reduction in HbA1c with no safety issues. However, the dipeptidyl peptidase-4 (DPP-4) inhibitor saxagliptin did not show a significant reduction in HbA1c.

Regarding the HbA1c reduction endpoint, our study found a statistically significant difference favoring SGLT2i over the placebo, both in the short and long term. This outcome was deemed clinically relevant based on previous pediatric T2DM studies, where HbA1c reductions of at least 0.5% were considered important, and around 0.3% reductions were considered borderline with uncertain clinical relevance [26, 31, 32, 38, 60–62]. Considering the acknowledged challenges in treatment adherence among children and young adults with T2DM [63, 64], we conducted a sensitivity analysis using only per-protocol data. The significant and clinically relevant outcome persisted even after excluding data following rescue and/or treatment discontinuation and after excluding patients with relevant protocol deviations in the short term. These findings aligned with pediatric pharmacokinetic–pharmacodynamic studies of SGLT2i [65, 66] and with efficacy and safety outcomes reported in adult populations [34]. The increase in HbA1c concentration observed in the placebo group across all individual trials was in accordance with previous studies in young patients with T2DM [67]. It is noteworthy that, although the proportion of patients with HbA1c < 7% at the study's end was significantly higher with SGLT2i in the short and long term, the sensitivity analysis considering only individuals with baseline HbA1c ≥ 7% resulted in the loss of statistical significance in the long term. This variability may be attributed to the 52.69% reduction in sample size in the sensitivity analysis or, more likely, to the aggressive nature of T2DM in pediatrics compared to adults. This is reflected in the progressive and accelerated *β*-cell failure observed in children and adolescents [68]. Besides, factors such as potential suboptimal compliance in younger populations, the development of drug resistance, lifestyle changes over time, and youth-specific variables may pose challenges in achieving treatment goals, all of which could limit the long-term efficacy of the drug [16]. Future studies should address these possibilities to enhance the long-term effectiveness of SGLT2i in managing T2DM in children, adolescents, and young adults.

Concerning the proportion of patients requiring rescue or discontinuing study medication due to lack of efficacy, no significant differences between SGLT2i and placebo were observed in the short term. It is crucial to emphasize that the T2NOW 2023 study may be the source of heterogeneity in this outcome. This trial encompassed a larger participant pool and was the only study that demonstrated significant and favorable results for the SGLT2i group in the short term. This discrepancy may be attributed to the differences in the T2NOW population compared to the DINAMO and Tamborlane et al. trials. The T2NOW study included participants primarily from Latin America and Asia/Pacific, introducing greater racial diversity, whereas the other studies predominantly enrolled participants from North America and Europe. Additionally, participants in the T2NOW trial exhibited a lower average body weight, a factor known to favor glycemic control [69]. Notably, nearly half of the individuals in the DINAMO trial were using insulin, which has been linked to nondurable glycemic control and is considered a marker of rapid deterioration of *β*-cell function [70]. In the long-term analysis, the SGLT2i group exhibited a lower and statistically significant proportion of patients requiring rescue or treatment discontinuation due to lack of efficacy. It is noteworthy that the T2NOW trial significantly influenced the long-term analysis, carrying a weight of 83.2%, resulting in a favorable outcome for the SGLT2i group, despite the pooled analysis showing no heterogeneity. Therefore, the different criteria that composed each of these studies pose a limitation on the analysis, especially since only one study had a large sample among the few available in scientific literature. Because of this, more studies with larger samples and diverse population groups are fundamental for a more complex and broader analysis that could consider how multiple variables, such as geographic location, body weight, and racial diversity, might affect the behavior of these drugs in pediatric and young adult populations with different characteristics.

Regarding blood pressure control, our findings did not reveal statistically significant differences in the change from baseline for both systolic and diastolic blood pressures between the SGLT2i group and the placebo group. This consistency was observed across all three trials but contrasts with studies in adults with T2DM [71]. While SGLT2i are known to induce osmotic diuresis, leading to a decrease in SBP in adults, the underlying mechanisms responsible for this effect remain complex. Prior studies in adults have proposed that the reduction in SBP may be attributed to both weight–loss-associated and weight–loss-independent pathways. The osmotic diuretic effect, driven by increased urinary glucose excretion, contributes to a negative energy balance, resulting in weight loss in some patients. The weight–loss-independent mechanism responsible for the reduction in SBP, which remains incompletely elucidated, is believed to involve diuresis leading to a contraction of circulating volume. This effect appears to surpass the activation of the renin–angiotensin–aldosterone system, suggesting a complex interplay of hemodynamic factors contributing to the observed decrease in SBP [71, 72]. In our meta-analysis, two individual trials (Tamborlane et al. and DINAMO) evaluated weight change but found no notable effects of SGLT2i. It remains unclear why reductions in blood pressure are not generally observed in pediatric patients. This raises intriguing questions about the precise interplay of factors influencing blood pressure regulation in pediatric and young individuals undergoing SGLT2i therapy. Such differences might reflect increased fluctuations in growth, maturation, and development during puberty.

Concerning safety outcomes, our data indicated no significant differences between SGLT2i and placebo in terms of any adverse event, severe side effects, and individual adverse effects during both short-term and long-term assessments. It is noteworthy that the T2NOW trial may again be the source of heterogeneity by including a population with a more favorable safety profile compared to DINAMO and Tamborlane et al. Specifically for serious adverse events, the DINAMO and Tamborlane et al. studies reported the serious adverse events encountered. In Tamborlane et al., [38] the events included depression, lower abdominal pain, spontaneous abortion, and two cases of hyperglycemia. In the DINAMO study, four events requiring hospitalization occurred, including a case of hyperglycemia and others of diverse nature [23].

Of particular note, the incidence of any type or severe hypoglycemia was comparable between the SGLT2i and placebo groups in both the short and long term. This outcome holds significance in the context of children and adolescents, as they face a higher likelihood of hypoglycemic events due to the disease's pathophysiology. This includes early insulin resistance and the more accelerated loss of pancreatic *β*-cell function over time when compared to adults with the same health condition. Additionally, difficulties in identifying symptoms and challenges in implementing nonpharmacological therapies, such as diet and exercise, contribute to the importance of this safety consideration [7, 15, 73, 74]. Given this favorable safety profile, more stringent HbA1c targets, such as levels below 6.5%, can be considered for these individuals during treatment [42]. However, these issues require further scientific investigation. It should be mentioned that although dapagliflozin, used in Tamborlane et al. and T2NOW, has a low propensity to cause hypoglycemia when used as monotherapy or in combination with most other glucose-lowering drugs, it carries an increased risk of this adverse event when combined with insulin [33]. In Tamborlane et al. (2022), for instance, hypoglycemia occurred in a third of patients taking dapagliflozin over 52 weeks, with almost all (10/13) also using insulin. In fact, the higher use of insulin in the dapagliflozin group versus the placebo group at baseline (SGLT2i 56.41% vs. placebo 39.39%) might have influenced the outcome [38].

The mechanisms linking SGLT2i to headaches are still under investigation. Potential explanations include the osmotic diuretic effect of the drug, leading to increased urine volume and potential dehydration, which can precipitate headaches. Additionally, the impact of SGLT2i on hypoglycemia, alterations in glucose metabolism, and electrolyte levels may also play a role [75]. Furthermore, SGLT2 inhibitors are known to increase the risk of DKA in adults [76]. If this risk is considered in children, DKA can lead to cerebral edema and presents with headaches [77]. In our analysis, we found no significant difference in the incidence of headaches between the SGLT2i and placebo cohorts in both the short-term and long-term follow-ups, with individual studies showing homogeneity. Although not statistically significant, the T2NOW trial reported headaches to be nearly three times more common in the SGLT2i group, constituting the most frequent adverse effect over 52 weeks (dapagliflozin 14.8%; placebo 5.3%). Nevertheless, these episodes were generally of mild intensity and subsequently resolved, with none considered serious or resulting in treatment discontinuation [24].

T2DM is commonly associated with urogenital infections due to factors such as hyperglycemia, treatment-induced glycosuria, and decreased humoral and cellular immunity. These conditions create a favorable environment for bacterial colonization and adherence to the uroepithelium [78–80]. A meta-analysis involving 52 randomized clinical trials and 36,689 patients [81] indicated a higher risk of UTIs attributed to dapagliflozin 10 mg compared with placebo. However, most reported episodes respond well to treatment with standard antibiotics [78]. Additionally, genital infections such as vulvovaginitis and balanitis tend to be more prevalent than UTIs [78]. In our study, there was no difference in the occurrence of UTIs between the SGLT2i and placebo groups, both in the short and long term. One possible explanation for this apparent lack of effect of SGLT2 inhibitors on UTIs in pediatric patients, despite observations in adults, could stem from differences in risk factors between the two populations. In adults, factors such as sexual activity, hormonal changes of menopause, pregnancy, neurogenic bladder dysfunction, and benign prostatic hypoplasia may add an increased risk of glycosuria [82]. However, it is important to note that other current pharmacologic options for T2DM in pediatric patients, such as GLP1 agonists, can also increase the risk of UTI [83]. Therefore, ongoing monitoring of these episodes is essential to ensure the safety of SGLT2i.

Another adverse effect of drug therapy for T2DM worth analyzing, especially in pediatric groups, is the rate of episodes of DKA during medication use. The present systematic review and meta-analysis found that there is no apparent effect of the use of SGLT2i on episodes of DKA in children and young adults with T2DM. This is an important difference compared to the adult population, as the use of these same medications in older individuals with the same condition carries the adverse effect of a greater risk of developing euglycemic DKA [84]. This difference may be explained by the very limited amount of data, which, although common in pediatric trials, may have prevented the observation of a relatively rare adverse event like DKA. Therefore, to properly confirm this difference in the risk of DKA with SGLT2i between children and adults, more studies with larger samples are needed.

A previous meta-analysis including 38 RCTs evaluated the efficacy and safety of SGLT2 inhibitors in adults with T2DM. In summary, this drug class showed significant improvements in HbA1c, FPG, body weight, and blood pressure. Specifically, all inhibitor subtypes had similar effects on body weight reduction. Regarding side effects, all inhibitors similarly increased the risk of genital infections [85]. In our meta-analysis for children, adolescents, and young adults, we found similar findings concerning HbA1c and FPG. However, we did not find a significant effect on weight reduction and blood pressure, which may be explained by growth changes, hormonal imbalances, and physiological insulin resistance during puberty. Altogether, these factors result in increased BMI and a poorer physiological profile in pediatric patients with T2DM compared to adults [11, 12].

Finally, our systematic review and meta-analysis suggest a reliable safety profile for SGLT2i across different time frames, aligning with the well-established safety profile of this drug class in adults [33]. However, our data have certain limitations that warrant consideration. First, the small number of studies with slight differences in design and small sample sizes, although a common feature of pediatric trials, limits the feasibility of detailed subgroup analyses. Second, the limited number of trials may raise concerns regarding the possibility of publication bias. Third, a substantial portion of the DINAMO trial was conducted during the COVID-19 pandemic, potentially impacting patients' ability to attend in-person consultations and reducing lifestyle efforts related to diet and exercise. Last, the short follow-up durations in studies by Tamborlane et al. and DINAMO prevented an extended long-term evaluation of SGLT2i efficacy, safety, prevention of cardiorenal complications, and nonglycemic outcomes. Consequently, future studies in pediatric patients are needed to elucidate the specific implications of SGLT2i in pediatric subgroups, such as those on insulin and at risk of hypoglycemia. Moreover, as the evidence for T2DM therapies in pediatric populations expands, future studies are needed to assess the impact of cointervention types (metformin vs. insulin vs. metformin + insulin), different types of SGLT2i, and the dose effects of SGLT2i. Ongoing trials on canagliflozin (NCT03170518) [86] and ertugliflozin (NCT04029480) [87] may help to answer additional questions. Nevertheless, the three analyzed RCTs had an unclear to low risk of bias.

## 5. Conclusion

To the best of our knowledge, this is the first systematic review and meta-analysis evaluating the efficacy and safety of SGLT2i in children, adolescents, and young adults with T2DM. Altogether, our results indicate that SGLT2i are the first oral glucose-lowering therapy since metformin to be clinically viable, effective, and safe for enhancing glycemic control in patients 10–17 years old and possibly similarly for those aged 18–24. Further randomized controlled trials with comprehensive subgroup analyses and extended long-term data are necessary to support our findings.

## Figures and Tables

**Table 1:** Study characteristics and baseline demographics.

Author and year		Tamborlane 2022	DINAMO 2023	T2NOW 2023	Total
Design (NCT)		RCT Phase III (NCT02725593)	RCT Phase III (NCT03429543)	RCT Phase III (NCT03199053)	N/A

Follow-up (weeks) mean		24 weeks	26 weeks	52 weeks	37.79 weeks

Intervention		Dapagliflozon 10 mg	Empagliflozin 10/25 mg	Dapagliflozin 5/10 mg	N/A

Number of patients, *N* (%)	SGLT2i	39 (54.17%)	52 (49.52%)	81 (51.59%)	172 (51.50%)
	Placebo	33 (45.83%)	53 (50.48%)	76 (48.41%)	162 (48.50%)

Female sex, *N* (%)	SGLT2i	24 (61.54%)	33 (63.46%)	49 (60.49%)	106 (61.63%)
	Placebo	19 (57.57%)	34 (64.15%)	44 (57.89%)	97 (58.08%)

Male sex, *N* (%)	SGLT2i	15 (38.46%)	19 (36.54%)	32 (39.51%)	66 (38.37%)
	Placebo	14 (42.43%)	19 (35.85%)	32 (42.11%)	65 (41.92%)

Age (years) mean ± SD	SGLT2i	16.1 ± 3.3	14.4 ± 1.9	14.4 ± 2	14.79 ± 2.42
	Placebo	16.2 ± 3.6	14.6 ± 1.8	14.7 ± 1.6	14.97 ± 2.28

Age group (years), *N* (%)	SGLT2i	10–15 years: 16 (41.02%) 16–17 years: 13 (33.33%) 18–24 years: 10 (25.65%)	10–14 years: 25 (48.08%) 15–17 years: 27 (51.92%)	10–14 years: 38 (46.91%) 15–17 years: 43 (53.09%)	10–17 years: 162 (94.19%) 18–24 years: 10 (5.81%)
	Placebo	10–15 years: 14 (42.42%) 16–17 years: 10 (30.30%) 18–24 years: 9 (27.28%)	10–14 years: 26 (49.06%) 15–17 years: 27 (50.94%)	10–14 years: 35 (46.05%) 15–17 years: 41 (53.95%)	10–17 years: 153 (94.44%) 18–24 years: 9 (5.56%)

Geographical region, *N* (%)	SGLT2i	North America: 16 (41.02%) Latin America: 7 (17.95%) Europe: 16 (41.03%)	North America: 36 (69.23%) South America: 9 (17.31%) Europe: 6 (11.54%) Asia: 1 (1.92%)	Latin America: 39 (48.15%) Asia/Pacific: 19 (23.46%) North America: 12 (14.81%) Europe: 11 (13.58%)	North America: 64 (37.21%) Latin America: 55 (31.98%) Europe: 33 (19.19%) Asia/Pacific: 20 (11.62%)
	Placebo	North America: 16 (48.48%) Latin America: 9 (27.27%) Europe: 8 (24.25%)	North America: 34 (64.15%) South America: 11 (20.75%) Europe: 7 (13.21%) Asia: 1 (1.89%)	Latin America: 23 (30.26%) Asia/Pacific: 23 (30.26%) Europe: 17 (22.37%) North America: 13 (17.11%)	North America: 63 (38.89%) Latin America: 43 (26.54%) Europe: 32 (19.75%) Asia/Pacific: 24 (14.82%)

Race, *N* (%)	SGLT2i	White: 28 (71.79%) Black or African American: 8 (20.51%) Native American or Alaska Native: 2 (5.13%) Other: 1 (2.57%)	White: 23 (44.23%) Black or African American: 19 (36.54%) American Indian or Alaska Native: 4 (7.69%) Asian: 2 (3.85%) Native Hawaiian or other Pacific Islander: 0 (0%) Other: 4 (7.69%)	White: 42 (51.85%) Asian: 18 (22.22%) American Indian or Alaska native: 11 (13.58%) Black or African American: 7 (8.64%) Native Hawaiian/other Pacific Islander/Other: 3 (3.71%)	White: 93 (54.07%) Black or African American: 34 (19.77%) Other: 45 (26.16%)
	Placebo	White: 16 (48.48%) Black or African American: 10 (30.30%) Native American or Alaska Native: 3 (9.09%) Other: 4 (12.13%)	White: 29 (54.72%) Black or African American: 17 (32.07%) Asian: 3 (5.66%) American Indian or Alaska Native: 1 (1.89%) Native Hawaiian or other Pacific Islander: 1 (1.89%) All other:2 (3.77%)	White: 32 (42.10%) Asian: 24 (31.58%) American Indian or Alaska native: 12 (15.79%) Black or African American: 3 (3.95%) Native Hawaiian/other Pacific Islander/other: 5 (5.98%)	White: 77 (47.53%) Black or African American: 30 (18.52%) Other: 55 (33.95%)

Mean duration of T2DM (years) mean ± SD	SGLT2i	3.10 ± 2.67	N/A	2.3 ± 1.7	N/A
	Placebo	3.15 ± 3.05	N/A	2.5 ± 2.2	N/A
Duration of T2DM (years) mean ± SD	SGLT2i	<3 years: 22 (56.41%) 3–10 years: 15 (38.46%) >10 years: 2 (5.13%)	<1 year: 17 (32.69%) 1–3 years: 21 (40.38%) >3 years: 14 (26.93%)	<2 years: 35 (43.21%) ≥2–≤5 years: 41 (50.62%) >5 years: 5 (6.17%)	N/A
	Placebo	<3 years: 21 (63.64%) 3–10 years: 10 (30.30%) >10 years: 2 (6.06%)	<1 year: 18 (33.96%) 1–3 years: 24 (45.28%) >3 years: 11 (20.76%)	<2 years: 40 (52.63%) ≥2–≤5 years: 25 (32.89%) >5 years: 11 (14.48%)	N/A

BMI (Kg/m^2^) mean ± SD	SGLT2i	31.3 ± 7.5	35.54 ± 7.17	29.7 ± 5.6	31.83 ± 6.99
	Placebo	33.6 ± 8.8	36.07 ± 10.07	28.5 ± 6.9	32.02 ± 9.06
BMI *z* score mean ± SD or *N* (%)	SGLT2i	1.69 ± 0.91	>2–≤ 3 (Class 1 obesity): 21 (40.38%) >3 (Class 2–3 obesity): 26 (50%)	1.7 ± 0.7	N/A
	Placebo	1.84 ± 1.08	> 2 to ≤3 (Class 1 obesity): 17 (32.07%) >3 (Class 2–3 obesity): 27 (50.94%)	1.5 ± 0.8	N/A

Bodyweight (Kg) mean ± SD	SGLT2i	89.2 ± 25.7	98.66 ± 24.35	78.8 ± 18.1	87.16 ± 23.48
	Placebo	92.5 ± 31.9	98.38 ± 29.55	76.2 ± 23.1	86.78 ± 28.93

Blood pressure (mmHg) mean ± SD	SGLT2i	SBP: 119.4 ± 12.9 DBP: 73.4 ± 8.8	SBP: 120.23 ± 9.97 DBP: 72.03 ± 8.38	SBP: 113.3 ± 12.1 DBP: 70.3 ± 8.1	SBP: 116.78 ± 12.08 DBP: 71.53 ± 08.39
	Placebo	SBP: 118.2 ± 15.2 DBP: 75.8 ± 7.6	SBP: 118.13 ± 11.85 DBP: 72.22 ± 9.27	SBP: 112.8 ± 11.2 DBP: 71.7 ± 9.3	SBP: 115.64 ± 12.53 DBP: 72.71 ± 09.06

HbA1c concentration (%) mean ± SD	SGLT2i	7.95 ± 1.59	8 ± 1.29	8.22 ± 1.46	8.09 ± 1.44
	Placebo	7.85 ± 1.19	8.05 ± 1.23	7.96 ± 1.63	7.97 ± 1.42

HbA1c concentration range, *N* (%)	SGLT2i	<6·5%: 5 (12.82%) ≥6·5%– <9%: 25 (64.10%) ≥9%–≤11%: 7 (17.95%) > 11%: 2 (5.13%)	<8.5%: 36 (69.23%) ≥8.5%: 16 (30.77%)	<6.5%: 11 (13.58%) ≥6.5–<8%: 26 (32.10%) ≥8–≤10.5%: 41 (50.62%) >10.5%: 3 (3.70%)	N/A
	Placebo	<6·5%: 2 (6.06%) ≥6·5%–<9%: 24 (72.73%) ≥9%–≤11%: 7 (21.21%) > 11%: 0 (0%)	<8.5%: 37 (69.81%) ≥8.5%: 16 (30.19%)	<6.5%: 13 (17.10%) ≥6.5–<8%: 30 (39.47%) ≥8–≤10.5%: 26 (34.21%) > 10.5%: 7 (9.22%)	N/A

Fasting plasma glucose (mg/dL) mean ± SD	SGLT2i	156.0 ± 55.7	154.4 ± 57.8	162 ± 64.8	158.34 ± 60.52
	Placebo	167.0 ± 63.2	158.6 ± 53.8	151.2 ± 57.6	156.84 ± 57.55

Tanner stage, *N* (%)	SGLT2i	Stage I: 0 (0%) Stage II: 1 (4%) Stage III: 3 (11%) Stage IV: 3 (11%) Stage V: 21 (75%) Not reported: 1 (4%)	Stage I: 0 (0%) Stage II–IV: 24 (46.15%) Stage V: 28 (53.85%)	Stage I: 1 (1.23%) Stage II: 11 (13.58%) Stage III: 11 (13.58%) Stage IV: 29 (35.80%) Stage V: 28 (34.58%) Not reported: 1 (1.23%)	Stage I: 1 (0.62%) Stage II–IV: 82 (50.62%) Stage V: 77 (47.53%) Not reported: 2 (1.23%)
	Placebo	Stage I: 0 (0%) Stage II: 2 (9%) Stage III: 2 (9%) Stage IV: 4 (18%) Stage V: 14 (64%) Not reported: 0 (0%)	Stage I: 0 (0%) Stage II–IV: 21 (39.62%) Stage V: 32 (60.38%)	Stage I: 1 (1.31%) Stage II: 7 (9.22%) Stage III: 10 (13.16%) Stage IV: 15 (19.74%) Stage V: 42 (55.26%) Not reported: 1 (1.31%)	Stage I: 1 (0.66%) Stage II–IV: 61 (40.40%) Stage V: 88 (58.28%) Not reported: 1 (0.66%)
Background antidiabetic drugs, *N* (%)	SGLT2i	Metformin only: 17 (43.59%) Insulin only: 7 (17.95%) Metformin + insulin: 15 (38.46%)	Metformin only: 26 (50%) Insulin only: 3 (5.77%) Metformin + insulin: 22 (42.31%) None (diet and exercise only, metformin not tolerated): 1 (1.92%)	Metformin only: 42 (51.85%) Insulin only: 10 (12.34%) Metformin + insulin: 29 (35.81%)	Metformin only: 85 (49.42%) Insulin only: 20 (11.63%) Metformin + insulin: 66 (38.38%) None (diet and exercise only, metformin not tolerated): 1 (0.57%)
	Placebo	Metformin only: 20 (60.61%) Insulin only: 5 (15.15%) Metformin + insulin: 8 (24.24%)	Metformin only: 28 (52.83%) Insulin only: 2 (3.77%) Metformin + insulin: 19 (35.85) None (diet and exercise only, metformin not tolerated): 4 (7.55%)	Metformin only: 39 (51.31%) Insulin only: 8 (10.53%) Metformin + insulin: 29 (38.16%)	Metformin only: 87 (53.70%) Insulin only: 15 (9.26%) Metformin + insulin: 56 (34.57%) None (diet and exercise only, metformin not tolerated): 4 (2.47%)

Metformin dose per day (mg) mean ± SD	SGLT2i	1666 ± 431	N/A	1450 ± 445	N/A
	Placebo	1625 ± 565	N/A	1539 ± 471	N/A

Insulin dose per day (IU) mean ± SD	SGLT2i	58.0 ± 42.7	N/A	38.9 ± 27.8	N/A
	Placebo	57.2 ± 48.1	N/A	37.4 ± 28.8	N/A

Abbreviations: BMI, body mass index; DBP, diastolic blood pressure; *N*, number of patients; N/A, not available; NCT, national clinical trial; RCT, randomized controlled trial; SBP, systolic blood pressure; SD, standard deviation; SGLT2i, sodium-glucose cotransporter 2 inhibitors; T2DM, type II diabetes mellitus.

**Table 2:** Risk of bias assessment.

Study	Bias from randomization process	Bias due to deviations from intended interventions	Bias due to missing outcome data	Bias in measurement of the outcomes	Bias in selection of the reported result	Overall risk of bias
Tamborlane 2022	Low	Low	Low	Low	Low	Low
DINAMO 2023	Low	Low	Low	Low	Low	Low
T2NOW 2023	Unclear	Unclear	Low	Low	Low	Unclear

**Table 3:** GRADE quality of evidence.

Certainty assessment							No. of patients		Effect		Certainty
No. of studies	Study design	Risk of bias	Inconsistency	Indirectness	Imprecision	Other considerations	SGLT2i	Placebo	Relative (95% CI)	Absolute (95% CI)	
The mean change in BMI *z* score from baseline (long)											
2	RCT	Serious^a^	Serious^b^	Not serious	Serious^c^	None	109	90	—	MD 0.03 lower (0.12 lower to 0.07 higher)	⨁◯◯◯ Very low

The mean change in HbA1c from baseline (long)											
3	RCT	Not serious	Not serious	Not serious	Not serious	None	172	162	—	MD 0.93 lower (1.36 lower to 0.49 lower)	⨁⨁⨁⨁ High

The mean change in FPG from baseline (long)											
3	RCT	Not serious	Not serious	Not serious	Not serious	None	168	161	—	MD 29.9 lower (44.65 lower to 15.14 lower)	⨁⨁⨁⨁ High

The proportion of patients achieving HbA1c < 7% at the end of the study (long)											
3	RCT	Not serious	Very serious^d^	Not serious	Not serious	None	37/138 (26.8%)	19/125 (15.2%)	RR 1.70 (1.02–2.84)	106 more per 1.000 (from 3 more to 280 more)	⨁⨁◯◯ Low

The rate of DKA (long)											
3	RCT	Not serious	Not serious	Not serious	Serious^e^	None	1/172 (0.6%)	2/162 (1.2%)	RR 0.61 (0.08–4.86)	5 fewer per 1.000 (from 11 fewer to 48 more)	⨁⨁⨁◯ Moderate

The proportion of patients requiring rescue or discontinuation of the study medication due to lack of efficacy (long)											
3	RCT	Not serious	Not serious	Not serious	Not serious	None	30/172 (17.4%)	44/162 (27.2%)	RR 0.64 (0.43–0.94)	98 fewer per 1.000 (from 155 fewer to 16 fewer)	⨁⨁⨁⨁ High

Abbreviations: BMI, body-mass index; CI, confidence interval; DKA, diabetic ketoacidosis; FPG, fasting plasma glucose; MD, mean difference; RCT, randomized controlled trial; RR, risk ratio.

^a^The unclear risk of bias in the T2NOW 2023 trial, which accounts for 55.2% of the weight of this outcome, is likely to reduce confidence in the estimate of effect.

^b^ *I^2^* = 44%.

^c^Due to the lack of statistical significance and wide confidence intervals of this outcome.

^d^The DINAMO 2023 trial, which represents 66.6% of the weight for this outcome, includes a population with HbA1c either above or below 7%. In contrast, the Tamborlane 2022 and T2NOW 2023 trials strictly evaluate this outcome in patients with HbA1c ≥ 7%. The overall outcome might be influenced by the inclusion of patients with a baseline HbA1c < 7%, as confirmed by the loss of statistical significance observed in the sensitivity analysis, which includes only the population with HbA1c ≥ 7%.

^e^The very limited sample size may have prevented the assessment of this relatively rare adverse event with reliable statistical power.

## Data Availability

The primary data that support the findings of this study are derived from published literature, and the relevant citations are provided in the references section. The raw datasets analyzed and collection forms are available from the corresponding author upon reasonable request.

## Supporting Information

Supporting Information The supporting information contains the following information: Complete search strategy; Table S1. Definitions used in the individual studies. Figure S1. Mean change in HbA1c from baseline—short follow-up. Figure S2. Mean change in HbA1c from baseline on short follow-up—sensitivity analysis excluding data following rescue and/or treatment discontinuation. Figure S3. Mean change in HbA1c from baseline on short follow-up—sensitivity analysis excluding patients with relevant protocol deviations. Figure S4. The proportion of patients achieving HbA1c < 7% at the end of the study—short follow-up. Figure S5. The proportion of patients with HbA1c ≥ 7% at baseline achieving HbA1c < 7% at the end of the study—short follow-up. Figure S6. The mean change in FPG from baseline—short follow-up. Figure S7. The proportion of patients requiring rescue or discontinuation of study medication due to lack of efficacy. Figure S8. The mean change in BMI *z* score from baseline—short follow-up. Figure S9. The mean change in SBP from baseline—short follow-up. Figure S10. The mean change in DBP from baseline—short follow-up. Figure S11. The rate of any adverse effect—short follow-up. Figure S12. The rate of serious adverse effects—short follow-up. Figure S13. The rate of adverse events leading to discontinuation of the study. Figure S14. The rate of any hypoglycemia event. Figure S15. The rate of severe hypoglycemia event. Figure S16. The rate of genital infections. Figure S17. The rate of ketoacidosis. Figure S18. The rate of headache. Figure S19. The rate of nasopharyngitis. Figure S20. The rate of vitamin D deficiency. Figure S21. The rate of UTIs. Figure S22. The mean change in HbA1c from baseline–SGLT2i subtypes. Figure S23. The rate of any adverse effect–SGLT2i subtypes.
