# Supplementary material for: Efficacy and Safety of SGLT2 Inhibitors in Pediatric Patients and Young Adults: A Systematic Review and Meta-Analysis of Randomized Controlled Trials
Source: Pediatr Diabetes. 2024 Oct 22;2024:6295345. doi: 10.1155/2024/6295345 (PMC12017006; doi:10.1155/2024/6295345)

Complete search strategy:

Pubmed: (((pediatric*[Title] OR young[Title] OR younger[Title] OR children[Title] OR child[Title] OR infant*[Title] OR adolescent*[Title]) AND (diabetes[Title] OR diabetic[Title] OR diabetal[Title] OR "diabetes mellitus"[Title] OR "type 2 diabetes mellitus"[Title] OR "type II diabetes mellitus"[Title] OR "type 2 diabetes"[Title] OR "type II diabetes"[Title] OR "diabetes mellitus type 2"[Title] OR "diabetes mellitus type II"[Title] OR "diabetes type 2"[Title] OR "diabetes type II"[Title])) AND (SGLT2[Title] OR SGLT-2[Title] OR "sodium-glucose cotransporter 2"[Title] OR "sodium-glucose cotransporter-2"OR "sodium-glucose co-transporter 2"[Title] OR "sodium-glucose co-transporter-2"[Title] OR "sodium-glucose transport protein 2"[Title] OR dapagliflozin[Title] OR empagliflozin[Title] OR canagliflozin[Title] OR sotagliflozin[Title] OR ipragliflozin[Title] OR luseogliflozin[Title] OR tofogliflozin[Title] OR bexagliflozin[Title] OR ertugliflozin[Title])) NOT ("type I diabetes"[Title] OR "type 1 diabetes"[Title] OR "diabetes type I"[Title] OR "diabetes type I"[Title])

Embase: (pediatric* OR young OR younger OR children OR child OR infant* OR adolescent*):ti AND ((diabetes OR diabetic OR diabetal OR “diabetes mellitus” OR “type 2 diabetes mellitus” OR “type II diabetes mellitus” OR “type 2 diabetes” OR “type II diabetes” OR “diabetes mellitus type 2” OR “diabetes mellitus type II” OR “diabetes type 2” OR “diabetes type II”):ti) AND ((SGLT2 OR SGLT-2 OR “sodium-glucose cotransporter 2” OR “sodium-glucose cotransporter-2” OR “sodium-glucose co-transporter 2” OR “sodium-glucose co-transporter-2” OR “sodium-glucose transport protein 2” OR dapagliflozin OR empagliflozin OR canagliflozin OR sotagliflozin OR ipragliflozin OR luseogliflozin OR tofogliflozin OR bexagliflozin OR ertugliflozin):ti) NOT (("type I diabetes" OR "type 1 diabetes" OR "diabetes type I" OR "diabetes type I"):ti)

Cochrane: (pediatric* OR young OR younger OR children OR child OR infant* OR adolescent*):ti AND (diabetes OR diabetic OR diabetal OR “diabetes mellitus” OR “type 2 diabetes mellitus” OR “type II diabetes mellitus” OR “type 2 diabetes” OR “type II diabetes” OR “diabetes mellitus type 2” OR “diabetes mellitus type II” OR “diabetes type 2” OR “diabetes type II”):ti AND (SGLT2 OR SGLT-2 OR “sodium-glucose cotransporter 2” OR “sodium-glucose cotransporter-2” OR  “sodium-glucose co-transporter 2” OR “sodium-glucose co-transporter-2” OR “sodium-glucose transport protein 2” OR dapagliflozin OR empagliflozin OR canagliflozin OR sotagliflozin OR ipragliflozin OR luseogliflozin OR tofogliflozin OR bexagliflozin OR ertugliflozin):ti NOT ("type I diabetes" OR "type 1 diabetes" OR "diabetes type I" OR "diabetes type I"):ti

Web of science: pediatric* OR young OR younger OR children OR child OR infant* OR adolescent* (Title) AND diabetes OR diabetic OR diacetal OR “diabetes mellitus” OR “type 2 diabetes mellitus” OR “type II diabetes mellitus” OR “type 2 diabetes” OR “type II diabetes” OR “diabetes mellitus type 2” OR “diabetes mellitus type II” OR “diabetes type 2” OR “diabetes type II” (Title) AND SGLT2 OR SGLT-2 OR “sodium-glucose cotransporter 2” OR “sodium-glucose cotransporter-2” OR “sodium-glucose co-transporter 2” OR “sodium-glucose co-transporter-2” OR “sodium-glucose transport protein 2” OR dapagliflozin OR empagliflozin OR canagliflozin OR sotagliflozin OR ipragliflozin OR luseogliflozin OR tofogliflozin OR bexaglifloxin OR ertugliflozin (Title) NOT "type I diabetes" OR "type 1 diabetes" OR "diabetes type I" OR "diabetes type I" (Title)

Table S1: definitions used in the individual studies

| **T2DM** | HbA1c ≥6·5% and ≤11% obtained at screening visit | Insufficient glycemic control as measured by the central laboratory at Visit 1A: a. DINAMOTM: HbA1c ≥ 6.5% and ≤ 10.5% b. DINAMOTM Mono: HbA1c ≥ 6.5% and ≤ 9.0 | HbA1c ≥6.5% and ≤10.5% obtained during the 6-month screening period |
| --- | --- | --- | --- |
| **Hypoglycemia** | ADA and ISPAD | ADA | ADA and ISPAD |
| **Criteria for glycemic rescue with insulin or increased doses of insulin** | FPG >240 mg/dL based on: • SMBG for 3 consecutive days followed by a confirmatory central laboratory FPG, or • Single central laboratory FPG followed by a confirmatory central laboratory FPG | • Acute metabolic decompensation accompanied by significant symptoms (e.g., vomiting, dehydration, lethargy) and/or repeatedly elevated blood ketone values > 27.03 mg/dL • Sustained hyperglycemia during SBGM (80% of blood glucose tests for 1 week are > 300mg/dL if non-fasting, or > 200 mg/dL if fasting) • If on two successive occasions (separated by at least 4 weeks) HbA1c is ≥ 9.0% and an absolute increase of HbA1c ≥ 1% compared with the baseline value is observed (even in the absence of symptoms related to hyperglycemia and ketoacidosis) | Week 6 visit up to and not including Week 26 visit FPG >240 mg/dL based on: • SMBG for 3 consecutive days followed by a confirmatory central laboratory FPG, or • Single central laboratory FPG followed by a confirmatory central laboratory FPG  Week 26 visit up to and not including Week 52 visit FPG >180 mg/dL based on: • SMBG for 3 consecutive days followed by a confirmatory central laboratory FPG, or • Single central laboratory FPG followed by a confirmatory central laboratory FPG, or • A1C >8·0% |

*T2DM* Type 2 diabetes mellitus, ADA American Diabetes Association, *ISPAD* International Society for Pediatric and Adolescent *Diabetes, FPG* Fasting plasma glucose, *SMBG* self-monitored blood glucose

Figure S1: mean change in HbA1c (%) from baseline (short follow-up)


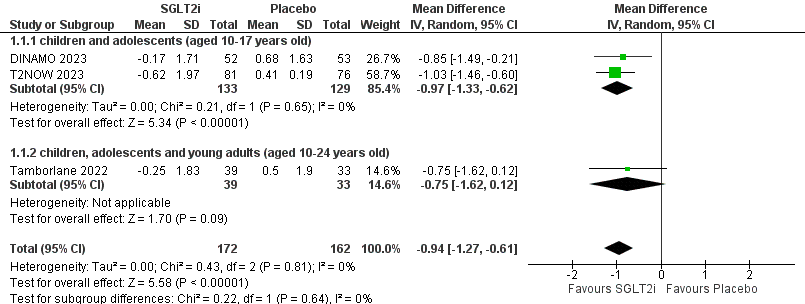


Figure S2: mean change in HbA1c (%) from baseline - sensitivity analysis excluding data following rescue and/or treatment discontinuation (short follow-up)


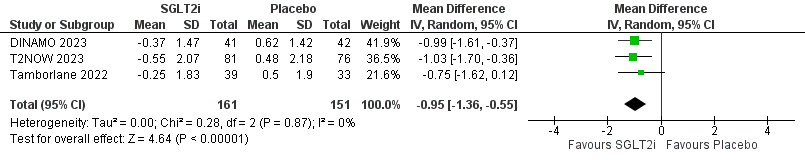


Figure S3: mean change in HbA1c (%) from baseline - sensitivity analysis excluding patients with relevant protocol deviations (short follow-up)


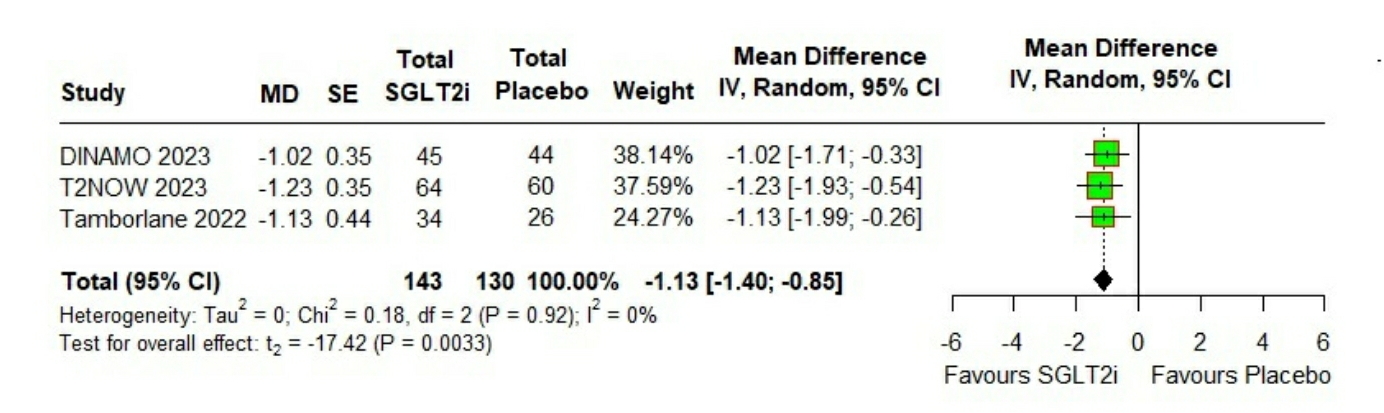


Figure S4: the proportion of patients achieving HbA1c < 7% at the end of the study (short follow-up)


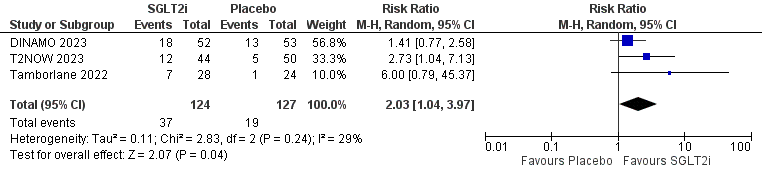


Figure S5: the proportion of patients with HbA1c ≥ 7% at baseline achieving HbA1c < 7% at the end of the study (short follow-up)


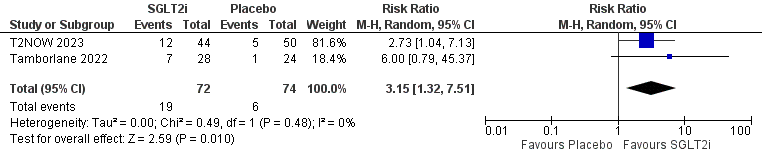


Figure S6: the mean change in FPG (mg/dL) from baseline (short follow-up)


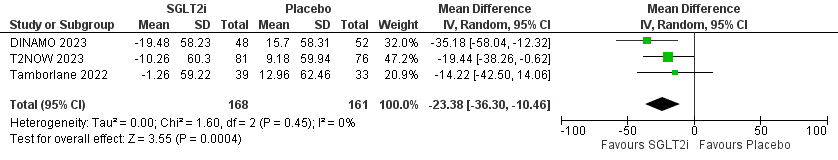


Figure S7: the proportion of patients requiring rescue or discontinuation of study medication due to lack of efficacy


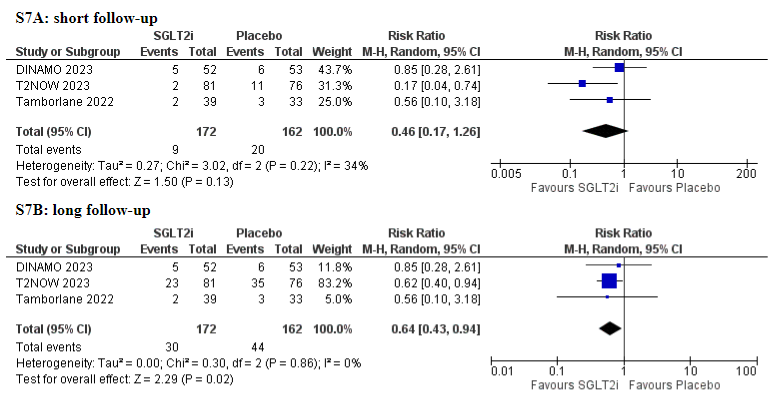


Figure S8: the mean change in BMI z score from baseline (short follow-up)


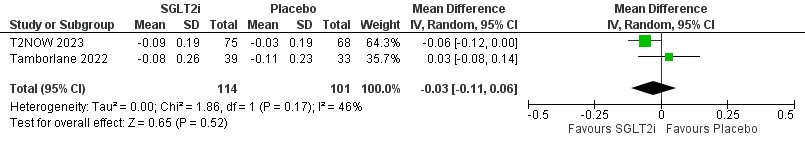


Figure S9: the mean change in SBP (mmHg) from baseline (short follow-up)


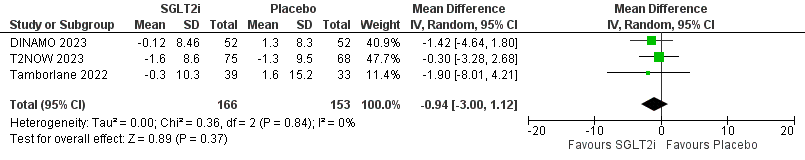


Figure S10: the mean change in DBP (mmHg) from baseline (short follow-up)


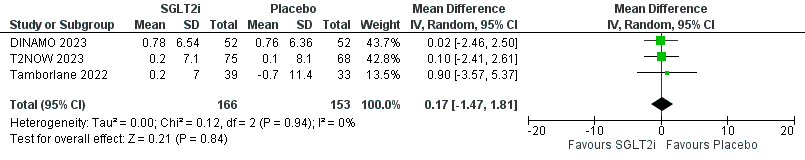


Figure S11: the rate of any adverse effect (short follow-up)


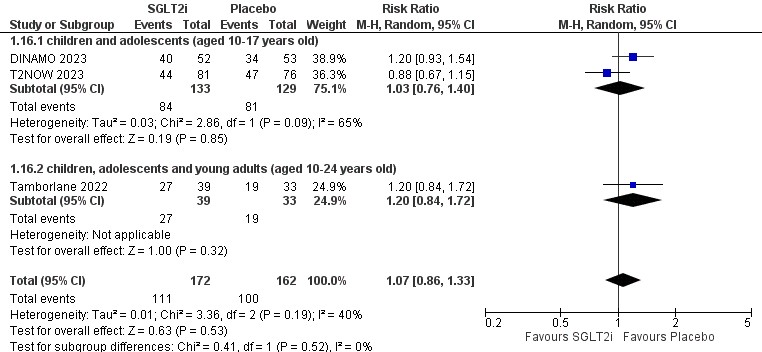


Figure S12: the rate of serious adverse effects (short follow-up)


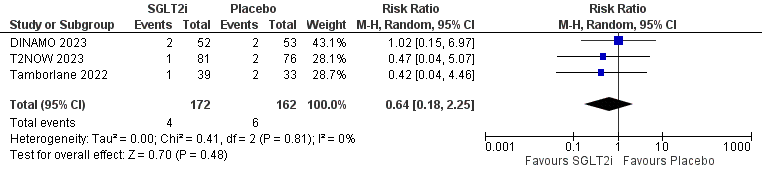


Figure S13: the rate of adverse events leading to discontinuation of the study


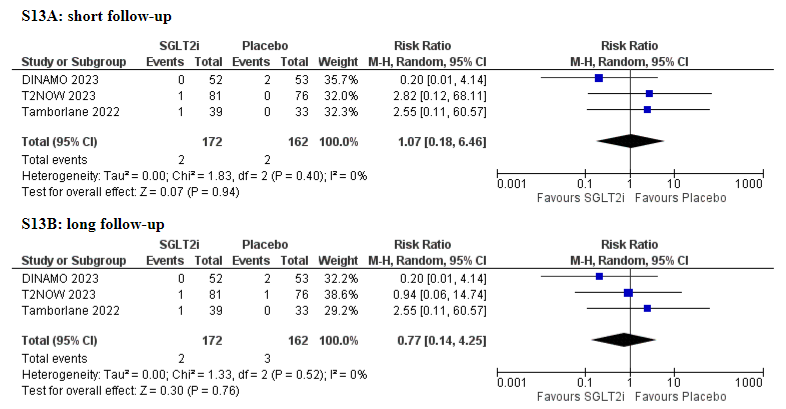


Figure S14: the rate of any hypoglycemia event


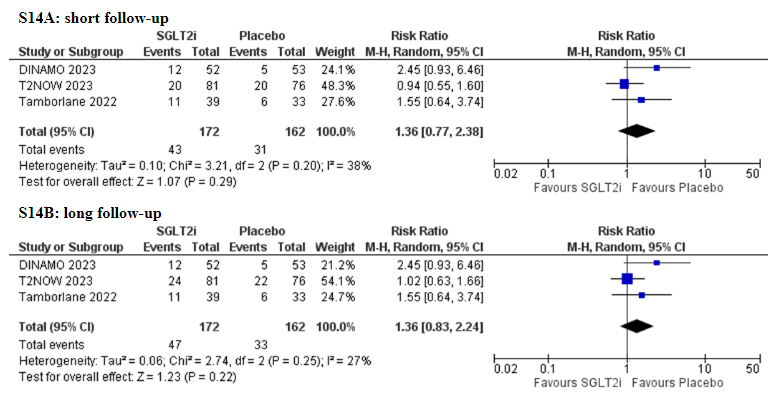


Figure S15: the rate of severe hypoglycemia events


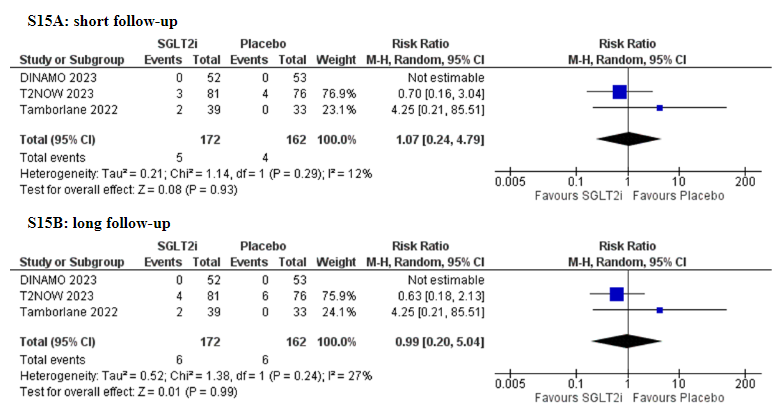


Figure S16: the rate of genital infections


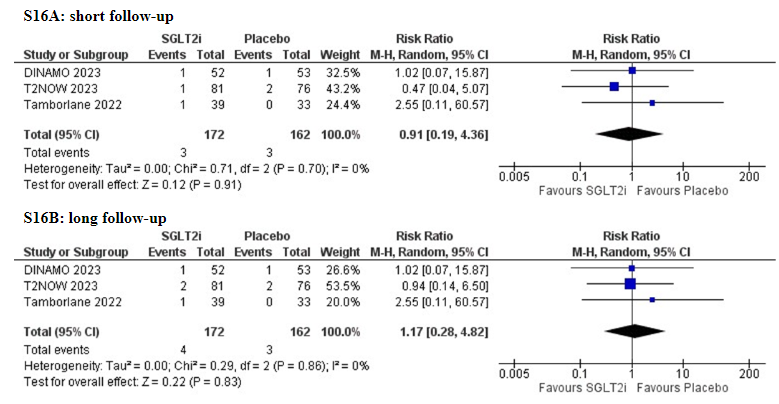


Figure S17: the rate of ketoacidosis


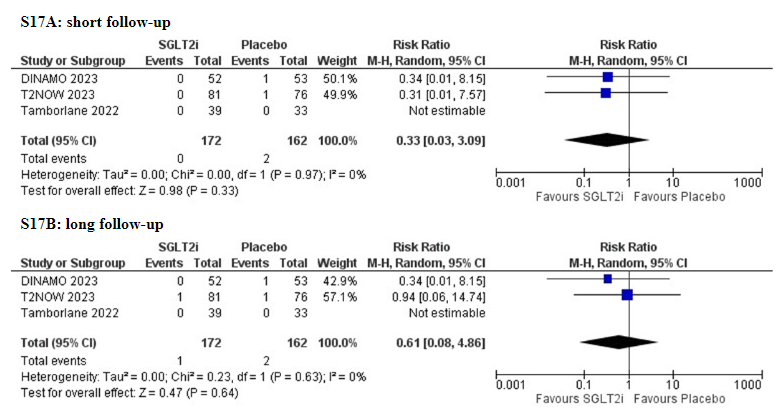


Figure S18: the rate of headache


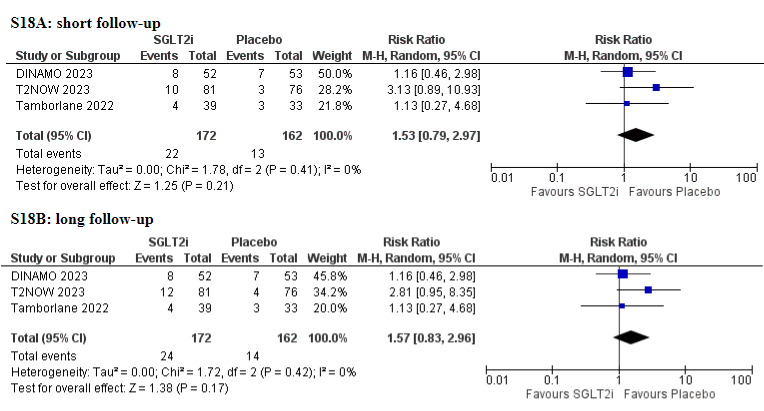


Figure S19: the rate of nasopharyngitis


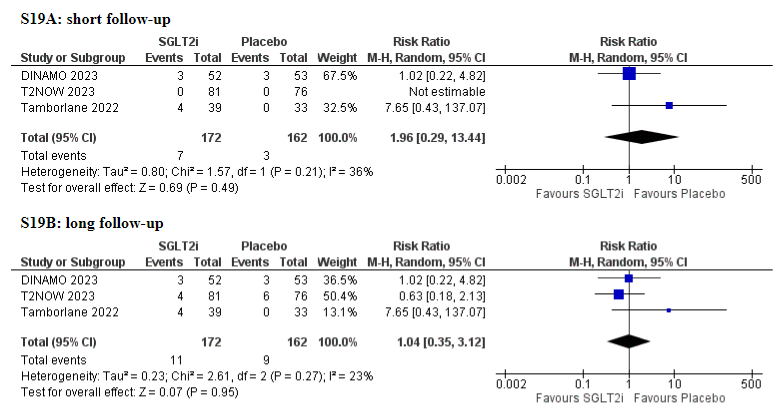


Figure S20: the rate of vitamin D deficiency


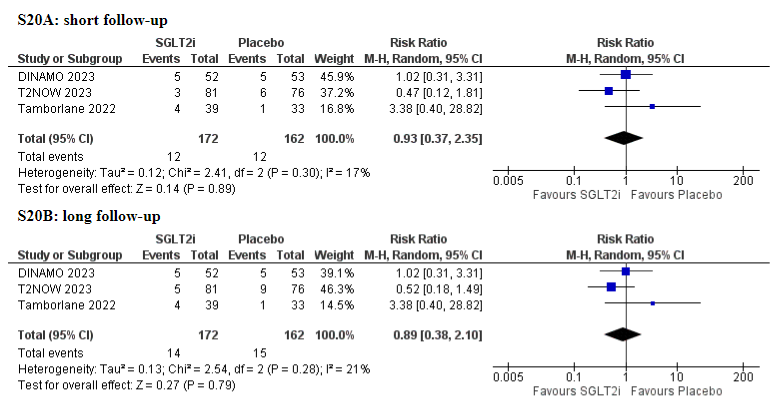


Figure S21: the rate of UTIs


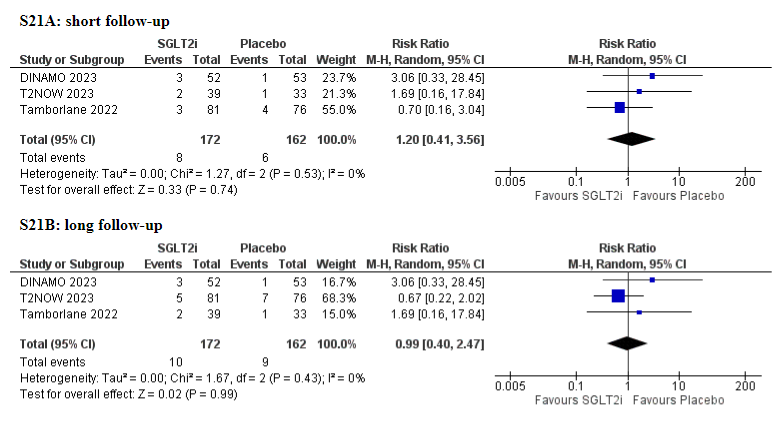


Figure S22: the mean change in HbA1c (%) from baseline – SGLT2i subtypes


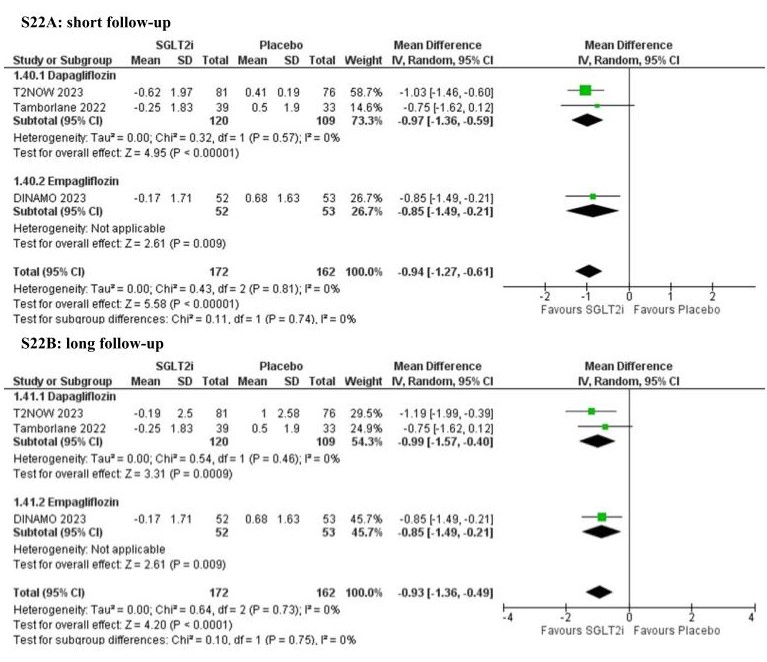


Figure S23: the rate of any adverse effect – SGLT2i subtypes


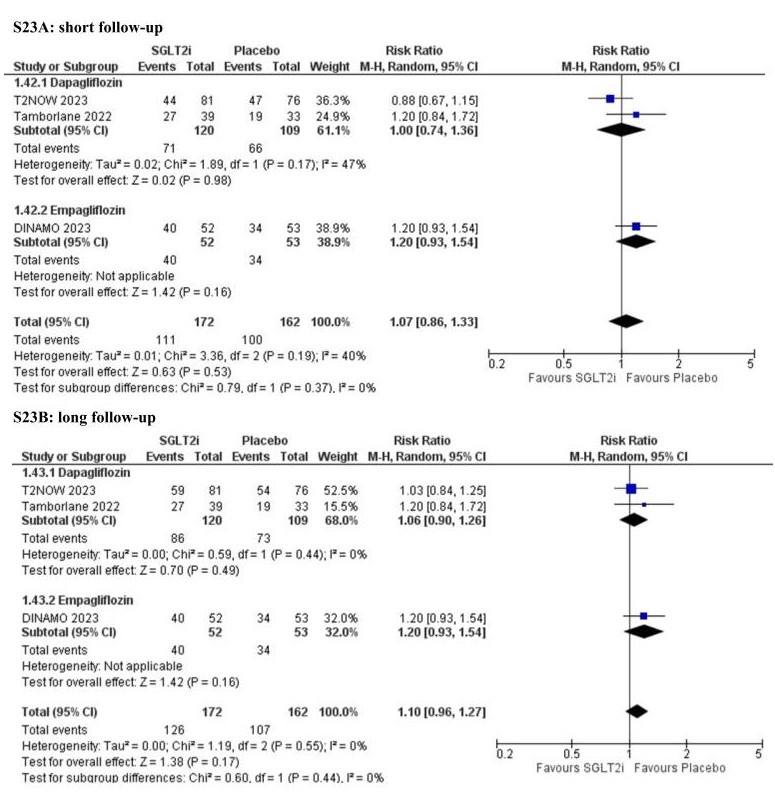

Supplement: Supporting Information — The supporting information contains the following information: Complete search strategy; Table S1. Definitions used in the individual studies. Figure S1. Mean change in HbA1c from baseline—short follow-up. Figure S2. Mean change in HbA1c from baseline on short follow-up—sensitivity analysis excluding data following rescue and/or treatment discontinuation. Figure S3. Mean change in HbA1c from baseline on short follow-up—sensitivity analysis excluding patients with relevant protocol deviations. Figure S4. The proportion of patients achieving HbA1c < 7% at the end of the study—short follow-up. Figure S5. The proportion of patients with HbA1c ≥ 7% at baseline achieving HbA1c < 7% at the end of the study—short follow-up. Figure S6. The mean change in FPG from baseline—short follow-up. Figure S7. The proportion of patients requiring rescue or discontinuation of study medication due to lack of efficacy. Figure S8. The mean change in BMI z score from baseline—short follow-up. Figure S9. The mean change in SBP from baseline—short follow-up. Figure S10. The mean change in DBP from baseline—short follow-up. Figure S11. The rate of any adverse effect—short follow-up. Figure S12. The rate of serious adverse effects—short follow-up. Figure S13. The rate of adverse events leading to discontinuation of the study. Figure S14. The rate of any hypoglycemia event. Figure S15. The rate of severe hypoglycemia event. Figure S16. The rate of genital infections. Figure S17. The rate of ketoacidosis. Figure S18. The rate of headache. Figure S19. The rate of nasopharyngitis. Figure S20. The rate of vitamin D deficiency. Figure S21. The rate of UTIs. Figure S22. The mean change in HbA1c from baseline–SGLT2i subtypes. Figure S23. The rate of any adverse effect–SGLT2i subtypes. [file 6295345.f1.docx]
